# ECG Recurrence Plot-Based Arrhythmia Classification Using Two-Dimensional Deep Residual CNN Features

**DOI:** 10.3390/s22041660

**Published:** 2022-02-20

**Authors:** Bhekumuzi M. Mathunjwa, Yin-Tsong Lin, Chien-Hung Lin, Maysam F. Abbod, Muammar Sadrawi, Jiann-Shing Shieh

**Affiliations:** 1 Department of Mechanical Engineering, Yuan Ze University, Taoyuan 32003, Taiwan; mathunjwabhekie@gmail.com; 2 AI R&D Department, New Era AI Robotic Inc., Taipei 10571, Taiwan; lotusytlin@calcomp.com.tw (Y.-T.L.); lance_lin@neweraai.com (C.-H.L.); 3 Department of Electronics and Electrical Engineering, Brunel University London, London UB8 3PH, UK; maysam.abbod@brunel.ac.uk; 4 Department of Bioinformatics, School of Life Sciences, Indonesia International Institute for Life Sciences, Jl. Pulomas Barat Kav 88, Jakarta 13210, Indonesia; muammar.sadrawi@i3l.ac.id

**Keywords:** electrocardiogram, arrhythmia, recurrence plot, deep residual convolutional neural network

## Abstract

In this paper, an effective electrocardiogram (ECG) recurrence plot (RP)-based arrhythmia classification algorithm that can be implemented in portable devices is presented. Public databases from PhysioNet were used to conduct this study including the MIT-BIH Atrial Fibrillation Database, the MIT-BIH Arrhythmia Database, the MIT-BIH Malignant Ventricular Ectopy Database, and the Creighton University Ventricular Tachyarrhythmia Database. ECG time series were segmented and converted using an RP, and two-dimensional images were used as inputs to the CNN classifiers. In this study, two-stage classification is proposed to improve the accuracy. The ResNet-18 architecture was applied to detect ventricular fibrillation (VF) and noise during the first stage, whereas normal, atrial fibrillation, premature atrial contraction, and premature ventricular contractions were detected using ResNet-50 in the second stage. The method was evaluated using 5-fold cross-validation which improved the results when compared to previous studies, achieving first and second stage average accuracies of 97.21% and 98.36%, sensitivities of 96.49% and 97.92%, positive predictive values of 95.54% and 98.20%, and F1-scores of 95.96% and 98.05%, respectively. Furthermore, a 5-fold improvement in the memory requirement was achieved when compared with a previous study, making this classifier feasible for use in resource-constricted environments such as portable devices. Even though the method is successful, first stage training requires combining four different arrhythmia types into one label (other), which generates more data for the other category than for VF and noise, thus creating a data imbalance that affects the first stage performance.

## 1. Introduction

Arrhythmia is a form of heart condition that is characterized by the rate or the rhythm of the heartbeat. The heartbeat can be faster than normal, or too slow, or have an irregular pattern. Tachycardia occurs when the heartbeat is too fast, and bradycardia is the heart disease that is associated with very slow heartbeats. The most commonly known cardiovascular diseases include types of arrhythmias such as ventricular fibrillation (VF), premature ventricular contraction (PVC), atrial fibrillation (AF), and premature atrial contraction (PAC), to name just a few. All genders and ethnicities are at risk of cardiovascular diseases in the United States [1]. There is a casualty related to heart disease every 36 s in the United States. America records about 655,000 deaths from heart diseases yearly, that is, one cardiovascular-related death in every four deaths [2]. The United States spent about USD 219 billion on heart disease-related costs each year in 2014 and 2015 [3].

Present electrocardiogram (ECG) arrhythmia recognition algorithms are dependent on the assessment of the morphology of a few ECG beats. The existing scientific literature outlines various studies of QRS complexes and long-duration ECG signal analysis. Studies have shown that QRS complexes are popular compared to long-duration ECG signal analysis for arrhythmia recognition [4]. However, more work is needed to find a more suitable segment size due to the variations in those features among individuals [5,6]. This is the motivation to investigate and find a solution to effectively diagnose cardiovascular diseases using more accurate short-duration continuous ECG beat signals. The proposed algorithm will simplify the calculation of ECG features and will ease the implementation of the solution in smartphones without cloud computing for real-time applications to monitor the health of patients.

The ECG signal is easily available and can be acquired using a number of mediums. The growing number of publications reflects the importance of its features, which include subjects such as arrhythmia, sleep, and deep learning (DL). A wide range of automatic ECG classification methods on signal processing techniques have been proposed over the years. These include wavelet transform [7,8], frequency analysis [9], support vector machines (SVMs) [10,11,12,13], artificial neural networks (ANNs) [11,13], decision trees [14], linear discriminant analysis [12], and Bayesian classifiers [13]. Recently, the most commonly pursued method involved the application of DL algorithms [15,16,17,18,19,20].

Essentially, time series represent data points in order of their occurrence. Time series analysis is one of the most common pattern recognition tasks in real life [21,22,23]. It includes examples such as biomedical signal analysis, biometrics, industrial devices, financial data, forecasting, and weather, to name just a few. Time series tasks come in different categories, which include classification, clustering, curve fitting, prediction and forecasting, and segmentation. Although this paper addresses the 2D classification problem, it employs time series data that are converted into 2D with the help of a recurrent plot (RP).

DL models, including convolutional neural networks (CNNs), have recently received increasing popularity. Unlike the traditional classification method, CNNs use 2D images and do not rely on feature extraction [24,25,26]. CNN models are capable of learning features and classifying them at the same time. Due to CNNs’ 2D data crunching, they make use of all data, including data that might otherwise be removed throughout noise reduction and feature extraction, which has a noticeable positive impact on their performance. In addition to the convolutional layers, several other processing units, such as a pooling process, sigmoid, a rectifying filter, and a normalization filter, are also responsible for learning a hierarchy from low-level to high-level features.

A CNN is a type of neural network that learns representations from data using a number of layers. A CNN consists of an input layer, a hidden layer (which includes a convolution layer), and an output layer. CNNs require little or no preprocessing compared to other classification networks due to their ability to learn and automatically optimize filters. A CNN is well known for understanding the image content and has been successfully applied to image classification, image segmentation, medical image analysis, natural language processing [27,28], financial time series analysis, recommendation systems [29], and brain–computer interfaces [30].

A residual neural network (ResNet) is another type of ANN which jumps over some layers using skip connections (double layer or triple layer). These skip connections include rectifiers and batch normalization [31] between them. A skip connection is added to mitigate the problem of degradation in the model, where adding more layers in a deep model causes a high number of training errors. ResNets were among the highest performers in the ILSVRC 2015 classification competition. The developers won first place in ImageNet detection, ImageNet localization, COCO detection, and COCO segmentation in the ILSVRC and COCO 2015 competitions. The ResNet architecture comes in several variations, based on the same principle, but with varying layers. For example, there are ResNet-18, ResNet-34, ResNet-50, ResNet-101, ResNet-110, and ResNet-152 [31].

For real-time arrhythmia detection, Fradi et al. [32] applied a multistage technique. In the first stage, R peak detection and noise removal were applied to the raw ECG data using a low-pass filter. In the second stage, a CNN-based fully connected layer was applied with a different network optimizer than in the first stage. Using a multistage process, Fradi et al. reported an accuracy of 99.37%, 99.15%, and 99.31% for training, validation, and testing, respectively. The authors in [33] used the RR intervals of the ECG as input for detecting the AF segment using an ANN for wearable ECG monitoring devices. According to their study, the model’s sensitivity, specificity, and accuracy were 99.3%, 97.4%, and 98.3%, respectively. Based on a deep 2D CNN, a model for ECG arrhythmias was proposed in [34]. A number of optimization strategies were applied during the normalization process, including Xavier initialization, data augmentation, and dropout. Ten-fold cross-validation using all the data as testing data was applied in that study to validate the classifier. The results achieved an average accuracy of 99.05%, with an average sensitivity of 97.85%. The authors in [35] developed an automatic arrhythmia classification strategy that uses an optimization-based deep CNN. The optimization algorithm was developed using the multi-objective bat and Rider optimization algorithms. The ECG wave and Gabor features were used as input features for arrhythmia detection in the deep CNN classifier. In [36], two classifiers for classifying heartbeat arrhythmia were proposed. The first classifier was designed based on a CNN and a long short-term memory (LSTM) network, whereas the other integrates the RR interval and higher-order statistics features with an LSTM model. Each model was trained separately on different datasets, and a weighted loss function was applied to each model to provide a high weight for insufficient data in a particular category. A meta-classifier was used to combine the predictions of the two classifiers to make a final prediction. Another CNN-LSTM classifier was used to verify the results.

The proposed study utilized ECG data acquired from four PhysioNet databases, namely, the MIT-BIH Atrial Fibrillation Database (AFDB), the MIT-BIH Arrhythmia Database (MITDB), the MIT-BIH Malignant Ventricular Ectopy Database (VFDB), and the Creighton University Ventricular Tachyarrhythmia Database (CUDB), to classify arrhythmia into six categories. The proposed approach has two stages. The first stage involves three classes, including VF, noise, and other, while the second stage classifies the categories contained by the class other in the first stage, which includes normal, AF, PAC, and PVC. During VF, the abnormal heart signals cause the ventricles to quiver. This may result in death; hence, VF requires immediate medical attention. Noisy signals can obscure the most important features of the signal, which, in turn, can lead to misclassification of the arrhythmia type. A careful approach is needed when dealing with data prior to classification, and identifying any potential interferences with an important feature can also contribute significantly. AF is characterized by the absence of a P wave, irregular RR intervals, and disorganized electrical impulses. A fast heart rate can also make atrial fibrillation appear more regular and harder to distinguish from other types of heart rhythm problems [37]. AF also increases the risk of heart failure by 1%, kidney problems by 0.5%, death by 0.4%, stroke by 0.3%, and coronary heart disease by 0.1% [38]. Despite the fact that PVC can occur in healthy individuals of any age, it is more common in the elderly and in men. Frequently occurring PVCs may indicate serious problems with the heart. Moreover, frequent PVCs may cause the heart to become less efficient, resulting in heart failure. PACs are similar to PVCs, but they are found in the atrium. They are caused by a premature contraction of the heart, resulting in an unsuccessful heartbeat. PACs in healthy patients do not indicate health risks, but they can trigger serious arrhythmias such as atrial fibrillation [39].

Previous research [40] by the authors has identified the types of arrhythmias discussed above, but there are some limitations that prevented the method from being applied to the intended purpose. The models require about 460 megabytes of memory space. By proposing a new model, it is aimed to improve upon our previous work. As a result, the proposed work improves the accuracy of the classifier for datasets with rhythm annotation in the first stage, resulting in a better overall classification accuracy compared to the previous study. In addition, the memory sizes of both the first and second stage classifiers are significantly reduced, enabling applying the proposed method to a broader range of devices that would not be suitable before due to the larger models in our previous work. In this work, it is aimed to develop a model that alerts patients to the possibility of cardiac risks so that they can consult with medical professionals for a further diagnosis and save their lives.

**Figure 1:**
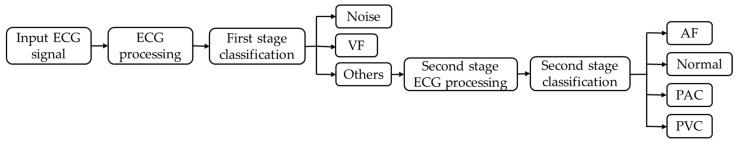
Diagram of the proposed approach.

The diagram shown in Figure 1 shows the procedure followed in the classification. The classification involves two stages. Input ECG data are fed into the classifier for preprocessing. Preprocessing begins with data segmentation in preparation to convert the time series signal into 2D images. The segmented 2 s segments are converted into 2D images using the recurrence plot method. The 2D images are then used as inputs in the first classification stage. There are three classes in the first stage, including noise, VF, and other. The other class of the first stage is further classified into four sub-classes by the second stage classifier. The second stage classifies ECG data into AF, normal, PAC, and PVC. If the data are classified as other in the first stage, they are sent to the second stage where they undergo further preprocessing. The second stage preprocessing involves the detection of the R peak and segmentation. One-second data before and after the R peak are combined to form a segment and converted to an RP for the second stage classification.

The one-dimensional (1D) ECG is converted to 2D images using an RP, allowing classifying the arrhythmias into one of six categories using a CNN. This study proposes an improved approach to classification in which two stages are used to improve accuracy. Data classification began by separating the labels of noise and VF, which required immediate attention. To begin with, it is necessary to determine the difference between VF and noise, since VF can imperil life, while noise can make the classification process more difficult. Due to the absence of R peaks in the segments detected in the first stage, the second stage cannot differentiate the aforementioned labels [41,42]. VF is caused when the heart’s organized electrical activity is disrupted, resulting in chaotic electrical impulses. The chaotic events result in a loss of the R peak since the myocardium’s action potentials cannot be synchronized. This is one of the reasons behind the proposal in implementing a two-stage classification system in this work. The remaining labels were segmented using the R-peak algorithm, and the segments were categorized into the different types of arrhythmias [43] and analyzed in the second stage. According to the results, the use of RPs and CNNs for arrhythmia discrimination appears to be feasible.

The following points summarize the contributions of this paper: (i) ECG arrhythmia detection is investigated by converting the time series ECG to 2D using the RP, which preserves all the useful features for ECG classification. This study introduces different layers of a ResNet in order to improve the performance of a previous study [40], achieving an average accuracy of 97.21% during the first stage and 98.36% during the second stage. (ii) The ResNet architecture reduces the model memory requirements by 5-fold, enabling implementation on mobile devices. (iii) By designing a low-memory classifier, a more dynamic system, which can adapt to changes in the database, can be created. Although the proposed classifier has more layers than the previous one, it is more computationally efficient and requires less training time. The remainder of the paper is structured as follows. Section 2 analyzes RPs to construct 2D segments of the ECG signal. In Section 3 and Section 4, methodology analysis is presented, including ECG data acquisition, ECG data preprocessing, ECG data classification, performance measures, the CNN classifier used in training, and results of the proposed methodology. Finally, Section 5 and Section 6 present a summary and conclusions.

## 2. Time Series to Recurrent Plots

The RP concept was introduced by Eckmann et al. [44] for visualizing the phase space trajectories, which are difficult to visualize in the time domain. This tool allows the exploration of the m-dimensional phase space trajectories by displaying their recurrence in two dimensions. It allows determining the point at which these trajectories return to a previous state. The main step in this visualization is the calculation of an N × N matrix. The numerical expression for an RP is defined according to Equation (1).

(1) $$R_{\mathrm{ij}}^{\mathrm{mn}}=\Theta \left(\varepsilon_{i}-∥x_{i}-x_{j}∥\right),{\;x}_{i}\in R^{m},\;\mathrm{ij}=1\ldots N.$$

where $\varepsilon_{i}$ is a cutoff distance; Θ is the Heaviside function; $x_{i}{\;\mathrm{and}\;x}_{j}\;$are the observed subsequences at both points i and j; || · || is the norm (Euclidian norm); and N is the number of states. Since $R_{\mathrm{ij}}$ = 1 (i = 1…N), the RP is composed of a black line along a diagonal line, which represents the identity line with an angle of Π/4. A trajectory reconstruction is performed using all recurrence points [45,46,47]. However, it is not possible for them to be rebuilt from a single occurrence point (i, j). In an m-dimensional time series j, whether the trajectory is almost identical to the time series is determined by the placement of black dots at coordinates; otherwise, white dots are placed. This method requires specification of the threshold parameter ε, used to binarize the R matrix, which is not easy to set.

This work adopts a modified version of an RP that utilizes color information. Color maps are used rather than Equation (1) to produce the image, which enables distances to be represented in color. This representation is known as unthresholded recurrence plot [48], as shown in Equation (2).

(2) $$R_{i,j}=∥x_{i}-x_{j}∥,\;\mathrm{ij}=1\ldots N.$$

**Figure 2:**
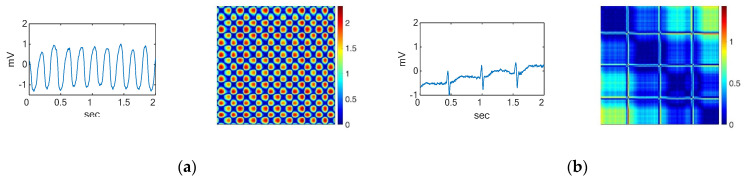

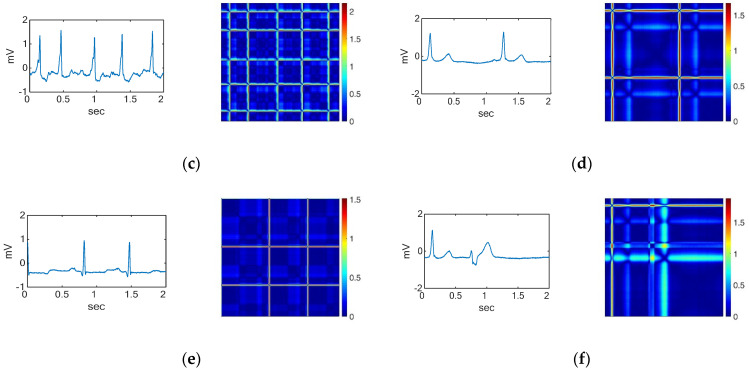
Waveforms and their corresponding RPs of the (**a**) VF rhythm, (**b**) noise, and the other four ECG arrhythmia types which need further classification including (**c**) AF, (**d**) normal, (**e**) PAC, and (**f**) PVC.

**Figure 3:**
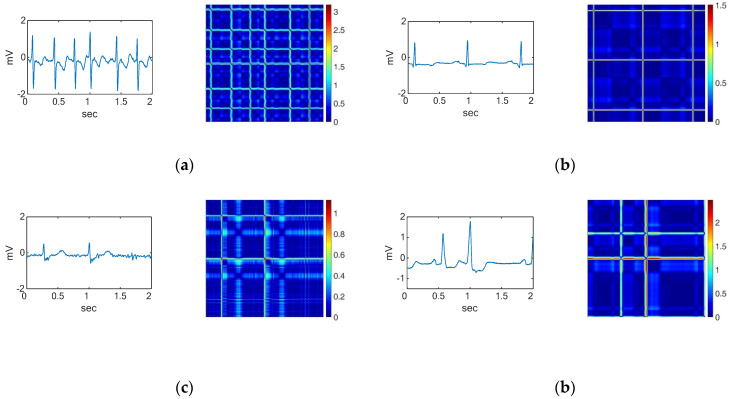
Waveforms and their corresponding RPs of (**a**) AF, (**b**) normal, (**c**) PAC, and (**d**) PVC, which are the four ECG arrhythmia types discriminated in the second stage.

Figure 2 and Figure 3 show examples of a typical modification of an RP for different types of signals. The RP images are created by defining a matrix of values between 0.0 and 1.0. Each row in the matrix represents a three-element RGB value, indicating the intensity of red, green, and blue. The converted three-axis signals of the RGB channel into an image that presents the contained information. The ECG signal is converted into 2D color images (Figure 2 and Figure 3) as input for the two-stage classifier. The ECG time series signals are converted into RGB images using the RP so that intensities can be exploited to improve the image resolution and accuracy of the model. Researchers have used an unthresholded RP to convert 1D signals into 2D color images [49,50,51,52].

## 3. Materials and Methods

In this study, a CNN was used to improve the classification of short-duration segments of ECG signals (2 s) [40]. The CNN was used for arrhythmia classification, involving two steps: preprocessing the ECG data and constructing the classifier. PhysioBank was used to resource data (PhysioNet) for the CNN model training, validation, and testing. Considering that ECG signals are 1D and the CNN model accepts 2D inputs, the ECGs were converted into 2D ECG images during the ECG data preprocessing step. An RP was applied to the data for transforming the ECG data and making it possible to perform the classification of the six arrhythmia categories in the CNN classifier step.

### 3.1. ECG Database

ECG signals were gathered from four publicly accessible datasets in PhysioNet [43]. Among the four datasets are the MIT-BIH Atrial Fibrillation Database (AFDB) [53], the MIT-BIH Arrhythmia Database (MITDB) [54], the MIT-BIH Malignant Ventricular Ectopy Database (VFDB) [55], and the Creighton University Ventricular Tachyarrhythmia Database (CUDB) [41]. A range of categories are provided by the MITDB, including normal, AF, PAC, and PVC. In this database are 48 ECG recordings of a half-hour length sampled at 180 Hz, obtained from 47 patients. Although the MITDB contains arrhythmia data with different categories, it does not provide enough data to satisfy the classification of all the categories under study. To satisfy the abovementioned arrhythmia categories, additional data for AF were obtained from the AFDB. This database includes 25 ECG recordings of subjects with atrial fibrillation. Out of the 25 ECG recordings of human subjects, only 23 are accessible for classification since 2 of the signals are only represented by rhythms and unaudited beats. The duration of each recording for this dataset is 10 h, and each recording is sampled at 250 samples per second. The data for the VF category were retrieved from the VFDB. This database includes 22 ECG recordings from subjects who experienced VF. The duration of the recordings is half an hour each, and the data are sampled at 250 samples per second. The data for the noise category were obtained from the CUDB. There are 35 ECG recordings in this database. The duration of the ECG recordings of the CUDB is about 8 min each, and the sampling frequency is 250 samples per minute.

### 3.2. ECG Data Preprocessing

The ECG recordings from the four datasets were sampled using different sampling frequencies. The MITDB was sampled at 360 Hz, while the AFDB, CUDB, and VFDB were sampled at 250 Hz. Records from all the datasets are available with beat and rhythm annotations, which were used for the isolation of the segments. A window of 2 s was considered (equivalence of 2 s = 720 samples for ECG from the MITDB and 500 samples for ECG from the AFDB, CUDB, and VFDB). The segments were annotated using the annotations made available in the databases.

An image serves as an input to the 2D CNN. Consequently, we converted each ECG segment into 2D images with the RP before classifying them. Even though the segment sizes are different due to the difference in the sampling frequencies for the datasets, the size of the resulting images is fixed to obtain the same size images. Figure 2 shows the ECG waveforms and their corresponding recurrence plot during the first stage of classification. The ECG waveforms for the second stage of classification and their corresponding RPs are shown in Figure 3.

### 3.3. Classification

The data segments were labeled in the first classification stage based on the beat and rhythm annotations provided in the records. Different annotation types were used to label segments in the first stage. During the first stage, other types of arrhythmias and VF were annotated with rhythm annotations, while noise was annotated with artifact annotations. An annotation was given to a segment if two-thirds of the data fell into that category in the first stage. Each segment of the data was annotated using the annotation used for the R peak in the middle of the segment during the second classification stage. Training, validation, and testing sets were randomly selected from the datasets (which made up 70%, 15%, and 15% of the total data used).

### 3.4. Performance Measures

To assess the performance of the CNN classifiers, we used accuracy (Acc), sensitivity (Sens), specificity (Sp), positive predictive value (PPV), F1-score (F1), and Cohen’s kappa (kappa). A total of six datasets were created from the four databases: the first stage was divided into VF and noise, while the second stage was divided into normal, AF, PAC, and PVC. Three subsets were randomly selected from the datasets: training, validation, and testing. For the purpose of assessing performance in multiclass classification, it was assumed that the proposed model is one that classifies samples into three classes, namely, A, B, and C. A confusion matrix for the model can be visualized as shown in Table 1. Analyzing performance involves comparing the following parameters.

1. Acc: This gives a matrix that describes how the model performs across all classes.

(3) $$\mathrm{Acc}\;=\frac{\mathrm{TP}+\mathrm{TN}}{\mathrm{TP}+\mathrm{TN}+\mathrm{FP}+\mathrm{FN}}\times 100\%$$

2. Sens: This gives the percentage of the true samples that were correctly detected by the algorithm.

(4) $$\mathrm{Sens}\;=\frac{\mathrm{TP}}{\mathrm{TP}+\mathrm{FN}}\times 100\%$$

3. Sp: This indicates the percentage of the samples that were correctly detected as negative segments and beats.

(5) $$\mathrm{Sp}\;=\frac{\mathrm{TN}}{\mathrm{TN}+\mathrm{FP}}\times 100\%$$

4. PPV: This is calculated according to Bayes’ theorem.

(6) $$\mathrm{PPV}\;=\frac{\mathrm{Sens}\times P}{\left(\mathrm{Sens}\times P+\left(1-\mathrm{Sp}\right)\times \left(1-P\right)\right)}$$

(7) $$P=\frac{\mathrm{TP}+\mathrm{FN}}{\mathrm{TP}+\mathrm{FP}+\mathrm{FN}+\mathrm{TN}}\;$$

5. F1: This gives the harmonic mean of the sensitivity and the positive predictive value.

(8) $$F1=2\times \frac{\mathrm{Sens}\times \mathrm{PPV}}{\mathrm{Sens}+\mathrm{PPV}}\times 100\%$$

6. Kappa:

(9) $$\mathrm{Kappa}\;=\frac{\mathrm{po}-\mathrm{pe}}{1-\mathrm{pe}}\times 100\%$$

(10) $$\mathrm{po}\;=\frac{\mathrm{TP}+\mathrm{TN}}{\mathrm{TP}+\mathrm{FP}+\mathrm{TN}+\mathrm{FN}}$$

(11) pe =(TN+FP)×(TN+FN)+(FN+TP)×(FP + TP)

7. True negative (TN): This represents the number of negative samples that were correctly predicted as negative by the model. TN is calculated for each of the three classes in the example in Table 1.

(12) $$\mathrm{TN}\left(A\right)={\;P}_{\mathrm{AB}}+P_{\mathrm{CB}}+P_{\mathrm{BC}}+P_{\mathrm{CC}}$$

(13) $$\mathrm{TN}\left(B\right)={\;P}_{\mathrm{AA}}+P_{\mathrm{CA}}+P_{\mathrm{AC}}+P_{\mathrm{CC}}$$

(14) $$\mathrm{TN}\left(C\right)={\;P}_{\mathrm{AA}}+P_{\mathrm{BA}}+P_{\mathrm{AB}}+P_{\mathrm{BB}}$$

8. False positive (FP): FP is the number of samples predicted by the model to be positive which, in fact, turned out to be negative.

(15) $$\mathrm{FP}\left(A\right)={\;P}_{\mathrm{AB}}+P_{\mathrm{AC}}$$

(16) $$\mathrm{FP}\left(B\right)={\;P}_{\mathrm{BA}}+P_{\mathrm{CB}}$$

(17) $$\mathrm{FP}\left(B\right)={\;P}_{\mathrm{BA}}+P_{\mathrm{CB}}$$

9. False negative (FN): FN is the number of positive samples that were incorrectly predicted as negative by the model. In multiclass classification, FNs are also calculated for each class.

(18) $$\mathrm{FN}\left(A\right)={\;P}_{\mathrm{BA}}+P_{\mathrm{CA}}$$

(19) $$\mathrm{FN}\left(B\right)={\;P}_{\mathrm{AB}}+P_{\mathrm{CB}}$$

(20) $$\mathrm{FN}\left(C\right)={\;P}_{\mathrm{AC}}+P_{\mathrm{BC}}$$

Multiclass classifications use the same TP as binary classifications do. However, true positives are calculated for each class in multiclass classification. The TPs of classes A, B, and C in Table 1 are represented, respectively, by the variables $P_{\mathrm{AA}}$, $P_{\mathrm{BB}}$, and $P_{\mathrm{CC}}$. The prevalence (P) is the percentage of the whole study population that has the target condition. P for the minority class in the population was used for PPV calculation. In an imbalanced classification problem, a minority class is a class with few examples. In this study, the first stage analyzed 29,217 images in 3 classes. There were 20,531 images categorized as other, 4256 images as noise, and 4430 images as VF. In the first classification stage, noise was a minority class with the lowest number of samples. The second stage analyzed 19,640 images, and 7228 of the images were in the normal ECG category, 6488 in the AF category, 2559 in the PAC category, and 3365 in the PVC category. According to this case, PAC is the minority class with the lowest number of samples. The PPV for the first and second stages was calculated according to the prevalence of noise and PAC, respectively. The po measure represents the proportion of units where there is agreement and is described in Equation (10). The pe measure represents the probability of random agreement.

For each of the models, all of the first and second stage data were tested in order to calculate the overall performance of the two-stage classifier. In the first stage, six classes (AF, noise, normal, PAC, PVC, and VF) were classified into noise, other, and VF. In the second stage, all images predicted as other by the first stage classifier were classified into four categories: AF, normal, PAC, and PVC. Then, the prediction confusion matrix was used to evaluate the two-stage classifier’s overall performance for the six-class classification using the defined metrics. For the two-stage classifier, PAC is the minority class as it has the lowest number of samples. PPV was calculated according to the prevalence of PAC in the two-stage classifier.

A receiver operating characteristic (ROC) curve is a visual representation of the false positive rates (sensitivity) and false negative rates (specificity). In the representation of the ROC curve, the *x*-axis shows the percentage of false positives, while the *y*-axis shows the percentage of false negatives. With ideal values provided, a point (0, 1) on an ROC curve indicates the test is more effective at separating cases from non-cases. The area under the ROC curve (AUC) is the area between the ROC and the axes, which can be anywhere between 0 and 1. An AUC that is closer to 1 indicates better test performances. When examining algorithm performance, the AUC metric is the proper tool since it does not rely on the prediction criteria. Classification models help to categorize observations into categories. Since the result of a classifier or diagnosis can be an arbitrary real value, a threshold value is required to determine the boundary between classes, and it is calculated from the ROC [56].

### 3.5. Two-Dimensional CNN Classifier

Our previous work monitored a number of classifiers and observed consistent experiences. In order to provide examples for discussion, we applied three CNN models successfully in the ImageNet Large Visual Perception Challenge (ILSVRC) [57,58] to the ECG arrhythmia classification. The ILSVRC is a competition for classifying objects in a set of images. Our previous work applied the AlexNet, VGG16, and VGG19 models. The AlexNet model took part, achieved first place in the competition of 2012, and was the first model to use a CNN model with the help of GPUs. The VGGNet model took part and achieved second place after GoogleNet in the same competition in 2014, and its structure is widely used in image recognition because of its simple structure. Although these models are successful in classifying arrhythmia with high accuracies, there remains a challenge in applying them for the intended purpose. Since the aim is to apply the classification capabilities of the models in mobile devices, the size of the model needs to remain small enough to be uploaded to the devices. Thus, further research is required to determine genetic models that recognize the type of arrhythmia present in RP segments with a lower memory requirement.

**Figure 4:**
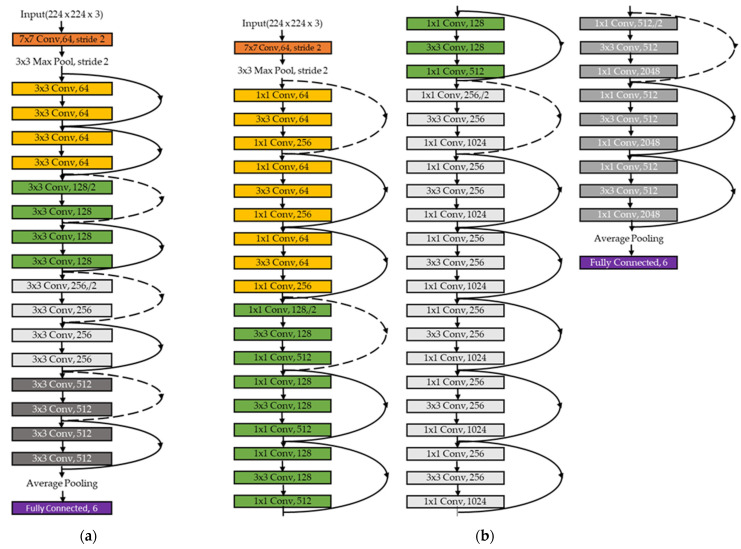
Architecture of the two adopted CNNs: (**a**) ResNet-18; (**b**) ResNet-50.

In this paper, the ResNet model was used to address the memory size problem we encountered with the other models and improve the performance of the first classification stage. The ResNet architecture is recommended for addressing the problems faced during the training of deeper networks. To find a suitable ResNet layer size that is effective in discriminating the six types of arrhythmias and requires less memory, five different ResNet architecture layer sizes were compared. Figure 4 shows the structure of the network architecture for ResNet (ResNet-18, Figure 4a; ResNet-50, Figure 4b). In Table 2, the five main features of each architecture are presented in detail. ResNet architectures begin with the initial convolution and maximum pooling using the 7 × 7 and 3 × 3 kernel sizes, respectively, as shown in Figure 4a,b. Afterwards, the first stage out of the four stages of the networks (represented in different colors) begins with two residual blocks containing two layers each for the shallow network. According to Figure 4, each pair of the 3-by-3 filters in both the 18 and 34 layers of the ResNet architecture has a shortcut connection added to it. For all shortcuts, identity mapping and zero padding are applied to increase dimensions, followed by a stride of 2. The deeper architectures including 50 layers, 101 layers, and 154 layers use their own building block due to concerns that more time is required to train them [31]. A stack of three layers is applied which includes 1 × 1, 3 × 3, and 1 × 1 filters, as shown in Table 2. The 1 × 1 layer reduces the dimensions and then increases them again, while the 3 × 3 layer remains as a bottleneck and has smaller dimensions [31]. As shown in Table 2, in the ResNet-18 architecture, the two-layer blocks are replaced with three-layer blocks to form the 50-layer network.

### 3.6. Training

The standard practice in [59] was observed in preparing images for the training procedure. We used color images generated by the RP. The RP images from the ECG time series were downscaled to 224 × 224 to reduce the training time [60]. Before feeding images to the network, the pixel means were computed from a fixed location over the training set and subtracted from each image, and then the network was trained using the centered RGB values of the pixels. The ECG signal was not normalized; instead, it was used to generate the 2D images which were fed to the network as training data. Data augmentation in this work was similar to that applied in [60], which alters the intensity of the RGB channels in the training images. Color models based on the RGB system combine red, green, and blue. In this data augmentation, image translations and horizontal reflections were generated to increase the size of the training dataset and reduce overfitting. Before activation and after every convolution, a batch normalization (BN) was applied [61]. BN refers to the process of re-centering and re-scaling the input layers so that the learning process takes less time and is more accurate. All residual nets were trained completely from scratch with weights initialized as shown in [58]. Network optimization was achieved using stochastic gradient descent (SGD) with a batch size of 256 samples. The network optimizer changed the attributes of the neural network, such as weights and the learning rate, to reduce losses. A batch size of 256 samples means that 256 samples will be used to estimate the error gradient before the model weights are updated. SGD is one of the commonly used algorithms for solving optimization problems [62]. Initially, 0.1 was set for the learning rate; when the error plateaued, it was divided by 10. As the learning rate was decreased during training, the accuracy of the model was improved, and overfitting was reduced. The weight decay was set to 0.0001, the momentum was set to 0.9, and no dropout was used [31,58]. The learning rate is a hyperparameter that determines the step size at each iteration while moving toward a minimum loss function. The learning rate influences the extent to which recently acquired information overwrites earlier information; thus, it represents the learning speed of the network. Weight decay helps constrict a network and therefore decrease its complexity by limiting weight growth. In this way, irrelevant components of the weight are suppressed by choosing the smallest ones. Momentum is a technique that is used along with SGD to improve the learning speed and accuracy. In addition to relying on the gradient of the current iteration, momentum also uses the gradient of the previous iteration in order to determine the direction of learning. The dropout technique was used to address the problem of overfitting. In dropouts, units and their connections are randomly removed from the network during training. In the ResNet-18 architecture, there are over 11 million trainable parameters, and there are over 23 million in the ResNet-50 architecture.

## 4. Results

### 4.1. Determining the Number of Layers in ResNet

We used the deep residual learning procedure implemented in [58]. As described in the previous section, we applied 0.9 for the momentum, and 0.0001 for the weight decay. We also utilized the weight initializer used in [57], and the batch normalization applied in [61]. The batch size was kept at 128 on one GPU (Nvidia Tesla K40 GPU). A learning rate of 0.1 was initially used, which was divided by ten every thirty-two thousand iterations. Similar data augmentation used in [63] for training was applied. The first stage of classification involved distinguishing between noise, VF, and other categories. In the first classification stage, 29,217 images were analyzed. A total of 20,531 images were categorized into the other category; 4256 images were classified as noise; and 4430 images were classified as VF. In the second classification stage, images were grouped into one of four datasets based upon the first stage. Normal, AF, PAC, and PVC datasets were included in the second stage. In the second round of classification, a total of 19,640 images were analyzed. A total of 7228 images constituted the normal set, 6488 constituted the AF set, 2559 constituted the PAC set, and 3365 constituted the PVC set. As a result of training and testing the image categories together, the predicted results were used to evaluate the predictive model. Based on the Acc, Sens, and Sp of the models, the performance of the first and second stages of classification was evaluated. To determine the ResNet layer size, a number of different layer sizes were trained, and the best ResNet was selected in terms of Acc and lower memory size. The number of layers for training was varied according to the available ResNet models from 18 to 152 layers [61].

Table 3 and Table 4 present the comparison of the accuracy for the ResNet models used to determine the layer size as well as the memory it requires in the two stages of classification. Table 5 and Table 6 present the comparison for the performance of the layers using Sens, and Sp in the two stages of classification. The chosen model for cross-validation and classification had the best performance along with the lowest memory requirement. Based on training, testing, and validating the various layer sizes, it appears that 18 and 101 layers are needed for the first and second stages of arrhythmia classification, respectively, when using ResNet algorithms. The training, validation, and testing results obtained after training the 18 layers of ResNet in the first classification stage were better than those obtained from our previous work [40]. As a result, the 18 layers of ResNet require less memory than the rest of the layer sizes, making them ideal for the first stage of classification. The results obtained from the 101 layers in the second classification stage were superior to those obtained from the 18, 34, 50, and 152 layers. The testing accuracy was 97.04% and 98.46% in the first classification and second classification stages using 18 and 101 layers, respectively. In the first and second stages of classification, 43 and 169 megabytes of memory were required for the 18 and 101 layers, respectively.

Since the aim is to apply the first and second stages of arrhythmia classification models in portable devices, it is very important to find an effective model that will be able to automatically discriminate between the six categories of arrhythmia and require a reasonably low memory size that will fit into the devices. Considering the fact that the memory size of the model is vital to the accomplishment of the intended purpose, it was decided to consider a layer size that will be able to meet both requirements of the models (good performance and less memory size) in the second classification stage. Then, the 50-layer ResNet was used for the second classification stage as a better alternative. The results presented in Table 4 show that the 50 layers have an accuracy of 98.15%, which is 0.31% less than the best testing accuracy (101 layers). The memory required for the 50 layers, on the other hand, is 75 megabytes less than that of the 101 layers.

### 4.2. Performance Evaluation

Presented in this section are the results of the three performance measures (training, validation, and testing) in the two stages of arrhythmia classification. For training, validation, and testing, the data were set at 70%, 15%, and 15%, respectively. The test results were used to assess the predictive ability of the models at both stages. The confusion matrix table, on the other hand, indicates the real classification results in the testing data versus the predicted results.

#### 4.2.1. Assessment of the First Stage of Classification

**Figure 5:**
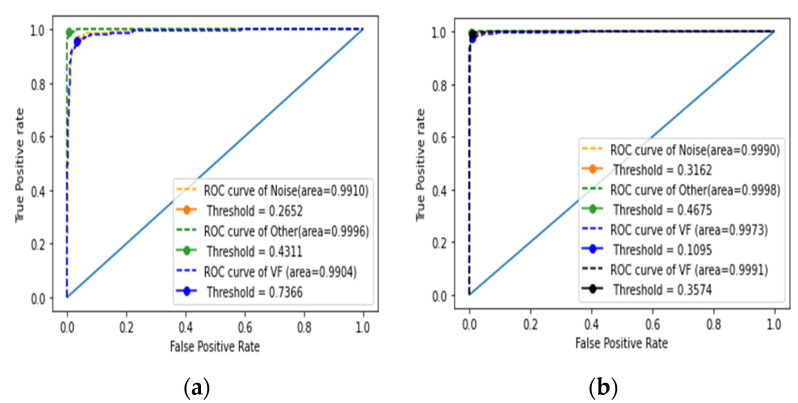
Performance metrics using the ROC curves: (**a**) first stage; (**b**) second stage.

To begin the classification process, the classifier was evaluated using three accuracy measures (training, validation, and testing accuracies). By utilizing the predicted results, Sp, Sens, and Acc values of the model were calculated based on the classification matrix. Table 7 reports the performance evaluation for the first stage of classification, which included cross-validation of input data for learning, validating, and testing. The first four columns of Table 7 show the representation of the cross-validation stage and the three sets of performance measures (training, validation, and testing accuracies) in the first stage of classification, respectively. The mean and standard deviation of the three performance measures are presented in the last row, which are 98.56 ± 0.16%, 96.76 ± 0.31%, and 97.21 ± 0.34%, respectively. Table 8 reports the performance measure for the first stage of classification cross-validation (5-fold) using sensitivity, specificity, and F1-score. The sensitivity in the first classification stage had a mean and standard deviation of 96.44 ± 0.47%, 93.77 ± 1.39%, and 99.27 ± 0.09% for the three datasets under classification (VF, noise, and other, respectively). According to the Sp, the first stage of classification reported a mean and standard deviation of 97.80 ± 0.76%, 99.22 ± 0.24%, and 98.97 ± 0.74% for the three datasets, respectively. The cross-validation in the first stage achieved a mean and standard deviation F1-score of 93.01 ± 1.10%, 95.46 ± 0.63%, and 99.42 ± 0.06%, respectively, for noise, other, and VF. Figure 5a shows the ROC curves for the first stage of classification. Table 9 shows the performance of the first stage classifier using the ROC curve. This performance shows the results of the 5-fold cross-validation for the three classes (noise, other, and VF). The threshold values to determine the boundary between the classes were also calculated and are presented in Table 9.

#### 4.2.2. Performance Evaluation of the Second Stage of Classification

According to Table 10, the performance of the second stage was evaluated using cross-validation, which took into account input data from three datasets (training, validation, and testing). The evaluation of the performance of the classifier used the means and standard deviations of the three sets of performance measures (training, validation, and testing accuracies), which were 98.72 ± 0.16%, 97.71 ± 0.16%, and 98.36 ± 0.16%, respectively. Table 11 reports the performance evaluation for the second stage of classification using Sens, Sp, and F1-score for the cross-validation (5-fold). According to the results of the second stage of classification, the means and standard deviations for sensitivity were 97.64 ± 0.42%, 99.65 ± 0.22%, 95.73 ± 1.11%, and 98.67 ± 0.48% for the four datasets (AF, normal, PAC, and PVC categories, respectively). According to the Sp, the second classification stage means and standard deviations for the four datasets (AF, normal, PAC, and PVC) were 98.90 ± 0.19%, 99.84 ± 0.10%, 99.52 ± 0.12%, and 99.52 ± 0.17%, respectively. The F1-score recorded means and standard deviations of 98.07 ± 0.08%, 99.18 ± 0.19%, 96.59 ± 0.46%, and 98.37 ± 0.18% for AF, normal, PAC, and PVC, respectively. Figure 5b shows the ROC curves for the second stage of classification. The performance of the second classifier using the AUC of the ROC curve for the second classifier is reported in Table 12. Performances are shown for the 5-fold cross-validation of the four classes (AF, normal, PAC, and PVC). The threshold values used to establish the boundary between the classes were also calculated and are presented in Table 12.

Table 13 reports the average accuracies for the first and second classification stages, where the achieved results during the 5-fold cross-validation were 96.49 ± 0.39% and 97.92 ± 0.30% for sensitivity, 98.66 ± 0.14% and 99.45 ± 0.04% for specificity, 93.29 ± 0.68% and 95.18 ± 0.37% for PPV, 95.96 ± 0.55% and 98.05 ± 0.19% for F1-score, and 95.28 ± 0.57% and 97.71 ± 0.20% for kappa, respectively.

In order to evaluate the overall performance of the proposed work, all classes were tested both in the first and second classification stages. There were three classes in the first stage, including noise, other, and VF. When predicted as noise or VF, the predictions were recorded under their respective categories, but if predicted as other, the RP image was sent to the second stage for further classification as AF, normal, PAC, or PVC. Table 14 shows the results of the procedure, with an accuracy of 94.85%, kappa of 94.44%, average sensitivity of 94.96 ± 2.94%, average specificity of 93.37 ± 7.31%, average F1-score of 94.05 ± 4.61%, and PPV of 93.37 ± 7.31%.

## 5. Discussion

Four datasets were used to train and test the procedure and different annotations were applied. Annotating the first stage data was based on the type of rhythm in two-thirds of the segment. In the second classification stage, the label for the R peak provided the segment annotation. One of the strengths of the proposed work is that it is able to classify arrhythmias with more than one annotation type, resulting in the ability to classify even more types of arrhythmias regardless of the type of annotation needed. Two seconds of data are required per segment, which may expose more than one arrhythmia type per segment. Because of this, the model is at high risk of misclassification; thus, more data are needed to balance the training data and enhance the possibility of a correct classification across all labels. The problem can be solved by adding more datasets with the same arrhythmia type in the future. All types of arrhythmias can be tested using both models with enough data. This study next implemented an AI system that is able to incorporate both models; thus, more testing was required. Having added more data and trained a more balanced dataset, both models were tested using the same datasets. Since the results of the second stage heavily depended on those of the first stage (other), it is important to increase the sensitivity of the first stage since the results for the second stage will be lower than those currently reported.

The results shown in Table 15 compare the proposed ECG diagnostic classifiers applied in this work with previous work [40]. The comparison includes the number of layers, the size of the model, the time taken for training the model, and the accuracies obtained using the same databases and number of training epochs. The proposed model’s results are compared to those reported in the previous work, confirming the effectiveness of the current approach, which can be utilized in mobile devices to classify a 2D arrhythmia based upon short durations of the arrhythmia.

In previous work, most of the work focused on recognizing the six categories of arrhythmia in two stages. Table 15 shows that the AlexNet model obtains the highest accuracy of 96.59% and 98.53%, respectively, in the first and second classification stages. Mobile devices, cloud computing, and telemedicine applications for real-time ECG arrhythmia analysis can potentially benefit from this accuracy in the classified types of arrhythmias. Despite the fact that the AlexNet model is efficient in ECG arrhythmia classification, it would be beneficial to reduce the model’s memory size.

In this study, the aim was to develop a classifier to enable edge computing on mobile devices. Since edge computing requires computation, data storage, and a close proximity to the source of data, powerful devices are also needed to make it possible. Mobile devices, for example, are equipped with storage and computing capabilities to make classification possible. This task requires both read-only memory (ROM) and random-access memory (RAM). Both permanent and non-permanent data are stored in the ROM. For instance, ROM is used for media, files, and games, while RAM is used for application and game execution, but once the applications are closed, the RAM is cleared.

Despite all mobile devices having both ROM and RAM, not all of them are capable of running certain data processing and classification due to the specific memory requirements. For the purpose of running software and graphic games, smartphones have RAM ranging from 2 GB to over 12 GB. Obviously, not all smartphone users require that much RAM. Different applications and games require different amounts of RAM memory, but larger amounts of RAM are necessary to run several programs at once smoothly. The two models (for the first and second stages) require 460 GB of storage space; thus, running them with other high graphic software and applications on a smartphone with limited RAM may negatively affect the user experience, resulting in many users not using the application.

The technique of image processing is largely determined by the processing capability of the device. As outlined above, random access memory and read-only memory are vital to a successful implementation of the method in mobile devices; thus, care must be taken when choosing the type of model to use. The financial constraints of many mobile device users limit their choice of devices, even though they would appreciate a variety of functions, including the one proposed in this study. Application developers should therefore consider these limitations when designing their applications. We can see from Table 15 that the proposed model requires less memory than the other models, allowing the method to be used in a wider range of devices and with other applications without changing or upgrading the RAM.

Table 15 shows that the proposed model has 10 and 42 more hidden layers than the previous method [40]. It is still able to archive a model that takes 185 and 134 megabytes less memory than the previous study. Additionally, this model produced networks that performed 0.62% better in the first stage and maintained a similar level of accuracy in the second stage. Despite the proposed model having up to 42 more layers than the previous work, the difference in the training time was less than an hour. The findings shown in Table 15 demonstrate an improvement over the previous study and contribute to the advancement of arrhythmia classification methods.

Table 16 compares the results of this study with those reported in other publications with respect to their respective arrhythmia categories, segment lengths, and average accuracies using various databases (mainly the MITDB). The Inception-ResNet-v2 network with RP images was used in Zhang et al. [64] as a classification method for cardiac arrhythmias. The CPSC database detected nine types of arrhythmias in their proposed work. Almost the same arrhythmia types except for VF and noise were used in their study with longer ECG segments. Compared to the two proposed stages, their work used a one-stage classification method. They reported average accuracies of 84.7%, 84.7%, and 84.4% for Sens, PPV, and F1-score, respectively. Ullah et al. [65] proposed a 2D CNN model for classifying eight types of arrhythmias. They reported 99.02% accuracy for classification using their model, which includes three convolutional layers, two downsampling layers, and a fully connected layer. Değirmenci et al. [66] classified five types of arrhythmias using a balanced distribution of ECG heartbeat images from the MITDB database, with an overall accuracy of 99.7%. Izci et al. [67] reported an accurate arrhythmia detection approach for five different types of arrhythmias that achieved 97.42% accuracy. As described in Le et al. [68], a multi-module recurrent convolutional neural network was used to fuse information from time series, spectrograms, and metadata modules for automatic ECG arrhythmia classification.

Overall, they reported an accuracy of 98.29% and an F1-score of 99.14%. Li et al. [69] presented a method for identifying VEB beats from artifacts by using a wavelet transform and a CNN. Their accuracy was 97.96%, and their F1-score was 84.94%. In [65,66,68], different algorithms were proposed to classify 2D ECGs with better accuracy than the proposed approach. Although most of them did not specify the segment length, it can be observed that they used shorter segments than the proposed work. Using longer segments in arrhythmia classification has the risk of exposing more than one label in the same segment, which may confuse the classifier. Moreover, different combinations of arrhythmia types were classified in the other works, which makes comparisons more challenging. However, results from similar studies may provide a basis for comparison.

Other studies also applied 1D-CNN techniques in their quest for effective arrhythmia diagnosis. In Chen et al.’s [70] study, they employed a CNN and an LSTM to detect six types of arrhythmias using 10 s ECG segments and attained 99.32%, 97.53%, and 96.66% accuracy for testing, Sens, and PPV, respectively. The MITDB was used to construct a 1D-CNN classifier for 17 types of arrhythmias by Yildirim et al. [71]. Based on 10 s ECG segments, they achieved an accuracy of 91.33%, 83.91%, and 91.33% for testing, sensitivity, and F1-score, respectively. In [72], two deep neural network models with residual convolutional modules and bidirectional LSTM layers were proposed to extract ECG features and concatenate them into input features for further training. The study analyzed ECG data from the CPSC database and obtained an overall F1-score of 80.6%. Yildirim et al. [73] used DBLSTM-based wavelet sequences to classify ECG signals. Their proposed study classified five types of arrhythmias based on the MITDB archive with an accuracy of 99.39%. Using time-varying features of ECG signals, Yao et al. [74] proposed a multiclass arrhythmia detection approach that integrates a CNN, recurrent cells, and attention modules. Their study achieved 81.2% classification accuracy. Fradi et al. [44] proposed a multistage 1D-CNN-based arrhythmia classifier that achieved an F1-score of 99% for five classes of arrhythmias.

In [75], ECG signal data were de-noised using a wavelet transform, and beat characteristics including RR intervals, morphological features, and statistical features were combined and used as input features for random forest classifiers, which achieved an average accuracy of 99.08%. El-Saadawy et al. [76] extracted features from ECG heartbeats, applied PCA to remove unwanted features, and classified the ECG signals based on an SVM, with an average accuracy rate of 88.7%. Sahoo et al. [77] used a PNN and radial basis function neural network (RBF-NN) to estimate six types of arrhythmias from an ECG signal, reporting an accuracy of 99.54% and 99.89%, respectively. Khairuddin et al. [78] implemented the Haar wavelet transform and k-nearest neighbor classifier to detect arrhythmias and achieved an average accuracy of 97.30%. The authors in [75,76,77] successfully used different machine learning methods to classify arrhythmias with high accuracy. Machine learning algorithms require less training and classification time, less processing power, and less data than CNNs but still take a lot of time during the preprocessing stage. CNNs may appear to require more resources and time, but once the classifier is ready, classification does not take much time.

Using only one beat as a basis for classification of arrhythmias has been studied with reasonable accuracy. When it comes to identifying arrhythmias that have abnormal heartbeats, it performs well. However, it cannot separate arrhythmias with abnormal rhythms. The proposed approach in this paper is well suited for these cases and can classify rhythm-based arrhythmias in the first stage and arrhythmias with abnormal heartbeats in the second stage.

Due to the types of arrhythmias examined in this study, this study is not comparable to other studies. The applied method is applied for the advancement of medical instrumentation (AAMI) arrhythmia types, which are the most studied arrhythmia types. This includes normal (N), ventricular ectopic (V), supraventricular ectopic (S), fusion (F), and unknown (Q). In Table 17, five types of arrhythmias are summarized where the AAMI arrhythmia beats are taken from the MITDB.

Since this work includes a different number of arrhythmia types, it is not suitable to be compared to other works. For a fair comparison, the proposed CNN model was compared with previous ECG arrhythmia classification works on the AAMI arrhythmia beats. Table 18 shows the cross-validation performance measurements of sensitivity, specificity, and F1 for each class (F, N, S, Q, and V). Additionally, Table 19 provides the results of a 5-fold cross-validation, including accuracy, kappa, PPV, and the averages of sensitivity, specificity, and F1-score. Using the AAMI arrhythmia beats, the proposed method achieved 98.21 ± 0.11% accuracy, 96.40 ± 0.54% average sensitivity, 96.89 ± 0.79% average specificity, 93.26 ± 2.61% PPV, 96.65 ± 0.19% F1, and 97.44 ± 0.15% kappa.

According to Table 20, compared to the accuracy from [15,68,79,80,81] the proposed model achieved a better classification performance, which indicates that using RP, RR detection, and the ResNet architecture can improve the classification accuracy of ECG arrhythmias. In their respective studies, the models in [82,83] performed better than the model in this study. The reported accuracy was 1.27% and 1.23% higher than that of the proposed work. This study’s F1-score and sensitivity were higher than those of [83]. The AAMI arrhythmia beat classification used in our study was based on the MITDB database. This database has a limited number of beats in other beat types, as shown in Table 17. Compared with two studies with better accuracy than this work, the nature of the beat types that were originally investigated in this study requires applying a longer segment length. By applying the same segment length to the AAMI, the achieved accuracy was reduced. In the future, further investigation will be conducted to determine whether an RP can be applied to AAMI arrhythmia classification with shorter ECG segment lengths. Additionally, additional databases will be included in order to improve the arrhythmia types with fewer data to improve the model’s accuracy.

**Figure 6:**
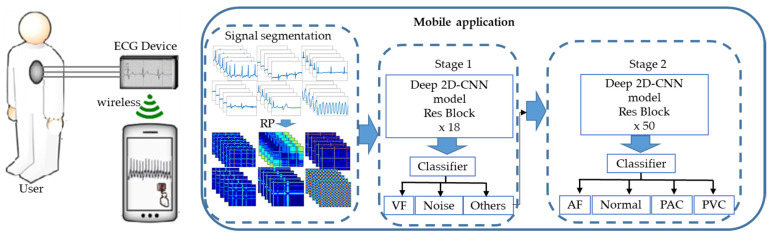
Illustration of how users can use the proposed short-duration ECG arrhythmia recognition.

Users can employ the proposed model as shown in Figure 6. The ECG device acquires and sends the ECG data wirelessly to the mobile phone for arrhythmia classification. When sent to the mobile phone, the data undergo preprocessing which includes signal segmentation and an RP to turn the signal into images for the trained classifier, which are also kept in the mobile device. Clinicians can use the results of the diagnosis as a reference for further analysis.

The proposed method utilizes two classifiers, one to classify the segments without the QRS complex, and the R peak classification. As a result, it may be impossible to apply the previously proposed solution to real-time signal processing in mobile devices due to the increased memory size. In that regard, the ResNet architecture [60] is the best model for reducing the memory size and providing the required learning capacity of a CNN, as shown in Table 15.

In the first and second stages of classification, accuracies of 97.21% and 98.36% were achieved, respectively, when using the 2D residual network model. Compared to our previous research [40], the proposed model improved the classification accuracy in the first stage of classification, resulting in a better overall accuracy than the previous study, and this solution can be easily implemented in mobile devices for real-time ECG arrhythmia classification.

In the proposed CNN approach, RPs are used as a representation of the ECG segment in the classification. In addition to large memory sizes for storage and usage, training large CNNs also requires long training times. Currently, the training time has been greatly reduced by using modern advanced hardware such as GPUs and supercomputers, reducing weeks of training for a very large model to days or even hours. The idea of skip connections used in highway networks is also applied in ResNet, which, in turn, helps to further preserve the training time. Table 15 shows a comparison to the AlexNet and VGG networks. ResNet is 20 and 8 times deeper and has a low computational complexity [37,58].

Despite the fact that this study shows interesting results on the specific types of arrhythmias under study, due to the lack of testing on random data and the lack of an accuracy threshold for obtaining random classification, it has some limitations. The results obtained thus far cannot be compared to random classification statistically.

In the proposed method, an RP is used to convert time series ECG signals into color images and use these as input for the classification of arrhythmias. The aim of this study was to develop a low-memory and effective ECG arrhythmia diagnostic model. However, color images were used instead of grayscale images to improve the accuracy of the model. Due to the fact that color images increase the network complexity, further research will be conducted to utilize grayscale images in order to reduce memory requirements and investigate the effect on accuracy.

## 6. Conclusions

This paper proposes a 2D residual CNN-based ECG RP-based arrhythmia classification method. PhysioNet provides access to four databases used to acquire ECG data for this study. A two-second segment of ECG data is segmented and converted into RP images. The RP images of the ECG are an effective representation of both the ECG beat and rhythm. In order to boost the classification performance, the application of the R peak recognition procedure was proposed for segmenting the second stage input data. For the development and testing of the proposed method, we utilized the MIT-BIH AFDB [55], the MIT-BIH Arrhythmia Database [56], the MIT-BIH Malignant Ventricular Ectopy Database [57], and the Creighton University Ventricular Tachyarrhythmia Database [58]. Based on 5-fold cross-validation, the accuracy of the two classifiers during the first and second classification stages was 97.21% and 98.36%, with a sensitivity of 96.49% and 97.92%, a positive predictive value of 95.54% and 98.20%, and an F1-score of 95.96% and 98.05%, respectively. Overall, the two-stage approach achieved an accuracy of 94.85%, sensitivity of 94.44%, specificity of 94.96, PPV of 94.05%, and kappa of 93.37%. At the first and second classification stages, better results were achieved in comparison to the previous work. As part of this study, a 5-fold improvement in memory requirements is demonstrated when compared with a previous study, making this classifier feasible for use in resource-constrained environments such as portable devices. Recurrence plots have been used in different areas of classification in the past, but more work is needed to support their use in CNNs. Four databases from PhysioNet with an RP were used for this study, but they have a limited number of arrhythmia categories. As a result, data imbalance affects the performance of the classifier. In future, more databases and detailed, broader studies will be examined to verify the effect of the RP method using a CNN.

## Figures and Tables

**Table 1:** Visualization of the confusion matrix.

		Predicted		
		A	B	C
Actual	A	$P_{\mathrm{AA}}$	$P_{\mathrm{BA}}$	$P_{\mathrm{CA}}$
	B	$P_{\mathrm{AB}}$	$P_{\mathrm{BB}}$	$P_{\mathrm{CB}}$
	C	$P_{\mathrm{AC}}$	$P_{\mathrm{BC}}$	$P_{\mathrm{CC}}$

**Table 2:** The structure of the two ResNet architectures that we used in this study. The two models comprised 18 and 50 layers.

Layer Name	Output Size	ResNet-18	ResNet-50
Conv 1	112 × 112	7 × 7, 64, stride 2	7 × 7, 64, stride 2
Conv 2_x	56 × 56	3 × 3 max pool, stride 2	3 × 3 max pool, stride 2
		$\left(\begin{array}{c} 3\times 3,\;64\\ 3\times 3,\;64 \end{array}\right)\times 2$	$\left(\begin{array}{c} 1\times 1,\;64\\ 3\times 3,\;64\\ 1\times 1,\;x256 \end{array}\right)\times 3$
Conv 3_x	28 × 28	$\left(\begin{array}{c} 3\times 3,\;128\\ 3\times 3,\;128 \end{array}\right)\times 2$	$\left(\begin{array}{c} 1\times 1,\;128\\ 3\times 3,\;128\\ 1\times 1,\;x512 \end{array}\right)\times 4$
Conv 4_x	14 × 14	$\left(\begin{array}{c} 3\times 3,\;256\\ 3\times 3,\;256 \end{array}\right)\times 2$	$\left(\begin{array}{c} 1\times 1,\;256\\ 3\times 3,\;256\\ 1\times 1,\;x1024 \end{array}\right)\times 6$
Conv 5_x	7 × 7	$\left(\begin{array}{c} 3\times 3,\;512\\ 3\times 3,\;512 \end{array}\right)\times 2$	$\left(\begin{array}{c} 1\times 1,\;512\\ 3\times 3,\;512\\ 1\times 1,\;x2048 \end{array}\right)\times 3$
	1 × 1	Average pool, 6-d fc, softmax	Average pool, 6-d fc, softmax

**Table 3:** Evaluating the first classification stage accuracy and model size to determine the layer size for ResNet.

ResNet Number of Layers	Training	Validation	Testing	Size (MB)
ResNet-18	98.77%	96.61%	97.04%	43
ResNet-34	98.12%	96.74%	97.45%	83
ResNet-50	98.40%	96.23%	96.53%	94
ResNet-101	98.55%	96.42%	97.26%	169
ResNet-152	98.60%	96.09%	96.72%	230

**Table 4:** Evaluation of the ResNet layers using the three performance measures (training, validation, and testing).

ResNet Number of Layers	Training	Validation	Testing	Size (MB)
ResNet-18	99.43%	94.06%	94.65%	43
ResNet-34	99.21%	95.59%	95.26%	83
ResNet-50	98.95%	97.22%	98.15%	94
ResNet-101	98.59%	97.91%	98.46%	169
ResNet-152	98.56%	97.62%	98.33%	230

**Table 5:** Evaluating the first classification stage using Sens and Sp to determine the ResNet layer size.

ResNet Number of Layers	Sens			Sp		
	Noise	Other	VF	Noise	Other	VF
ResNet-18	96.33%	92.96%	99.42%	97.16%	99.30%	99.28%
ResNet-34	95.93%	99.17%	94.35%	98.32%	99.00%	98.91%
ResNet-50	92.64%	98.58%	96.61%	97.03%	98.31%	99.33%
ResNet-101	93.20%	99.50%	96.89%	97.29%	99.37%	99.40%
ResNet-152	93.43%	98.75%	95.76%	97.34%	98.51%	99.17%

**Table 6:** Second stage Sens and Sp for the selection of the ResNet layers.

ResNet Number of Layers	Sens				Sp			
	AF	Normal	PAC	PVC	AF	Normal	PAC	PVC
ResNet-18	87.27%	99.67%	92.08%	98.49%	94.37%	99.84%	99.11%	99.46%
ResNet-34	88.35%	99.08%	96.04%	98.69%	94.83%	99.56%	99.55%	99.53%
ResNet-50	97.17%	99.67%	95.51%	98.49%	99.11%	99.80%	99.41%	99.28%
ResNet-101	98.34%	99.67%	96.31%	97.98%	99.22%	99.84%	99.59%	99.28%
ResNet-152	97.84%	99.42%	94.72%	98.99%	98.99%	99.73%	99.41%	99.64%

**Table 7:** First stage performance measure for the cross-validation (5-fold) using training validation and testing accuracies.

CV	Training	Validation	Testing
Mean ± SD	98.56 ± 0.16%	96.76 ± 0.31%	97.21 ± 0.34%

**Table 8:** First stage Sens, Sp, and F1-score for the 5-fold cross-validation.

	Noise			Other			VF		
CV	Sens	Sp	F1-Score	Sens	Sp	F1-Score	Sens	Sp	F1-Score
Mean	96.44	97.80	93.01	93.77	99.22	95.46	99.27	98.97	99.42
STD	0.47%	0.76%	1.10%	1.39%	0.24%	0.63%	0.09%	0.74%	0.06%

**Table 9:** Performance of the first stage cross-validation test using the AUC of the ROC curve.

CV	Noise		Other		VF	
	AUC	Threshold	AUC	Threshold	AUC	Threshold
Mean	0.991	0.273	0.999	0.536	0.991	0.626
STD	0.002	0.040	0.001	0.334	0.001	0.160

**Table 10:** Evaluation of the accuracy through training, validation, and testing with cross-validation (5-fold) in the second stage.

CV	Training	Validation	Testing
Mean ± SD	98.72 ± 0.16%	97.71 ± 0.16%	98.36 ± 0.16%

**Table 11:** Evaluation of the accuracy through Sens, Sp, and F1-score with cross-validation (5-fold) in the second stage.

	AF			N			PAC			PVC		
CV	Sens	Sp	F1-Score	Sens	Sp	F1-Score	Sens	Sp	F1-Score	Sens	Sp	F1-Score
Mean	97.64	98.90	98.07	99.65	99.84	99.18	95.73	99.52	96.59	98.67	99.52	98.37
STD	0.42%	0.19%	0.08%	0.22%	0.10%	0.19%	1.11%	0.12%	0.46%	0.48%	0.17%	0.18%

**Table 12:** Performance of the second stage cross-validation test using the AUC of the ROC curve.

CV	AF		Normal		PAC		PVC	
	AUC	Threshold	AUC	Threshold	AUC	Threshold	AUC	Threshold
Mean	0.998	0.234	0.999	0.624	0.998	0.144	0.999	0.264
STD	0.001	0.139	0.000	0.228	0.001	0.096	0.000	0.110

**Table 13:** Evaluation of the average accuracy through Sens, Sp, PPV, F1-score, and kappa with cross-validation in the first and second stages.

	Av Sens		Av Sp		PPV		Av F1-score		Kappa	
CV	1st Stage	2nd Stage	1st Stage	2nd Stage	1st Stage	2nd Stage	1st Stage	2nd Stage	1st Stage	2nd Stage
Mean	96.49	97.92	98.66	99.45	93.29	95.18	95.28	97.71	95.28	97.71
STD	0.39%	0.30%	0.14%	0.04%	0.68%	0.37%	0.57%	0.20%	0.57%	0.20%

**Table 14:** Overall performance of the two models through Acc, averages of Sens, Sp, PPV, and F1-score, and kappa.

Acc	Av Sens	Av Sp	Av PPV	Av F1-score	Kappa
94.85%	94.44 ± 2.94%	94.96 ± 7.31%	93.37 ± 7.31%	94.05 ± 4.61%	93.37%

**Table 15:** Comparison of the proposed approach with previous work.

Classifier	Layer Size	Model Size (MB)	Training Time (h)	Accuracy (%) First Stage	Accuracy (%) Second Stage
ResNet-18	18	43	6.26	97.21	94.65
ResNet-34	34	83	6.22	97.45	95.26
ResNet-50	50	94	6.32	96.53	98.36
ResNet-101	101	169	6.25	97.26	98.46
ResNet-152	152	230	6.26	96.72	98.33
AlexNet	8	228	5.37	96.59	98.53
VGG16	16	525	7.6	87.35	86.86
VGG19	19	545	7.75	81.01	94.09

**Table 16:** ECG arrhythmia classification evaluations.

Studies	Databases	No. of Classes	Segment Length(s)	Method	Acc (%)	Sens (%)	PPV (%)	F1-Score
Zhang et al. [64]	CPSC	9	5	Inception-ResNet-v2	N/A	84.7	84.7	84.4
Ullah et al. [65]	MITDB	8	N/A	2D CNN	99.02	N/A	N/A	N/A
Degirmenci et al. [66]	MITDB	5	N/A	CNN	99.70	99.70	99.22	N/A
Izci et al. [67]	MITDB	5	N/A	CNN	97.42	N/A	N/A	N/A
Le et al. [68]	MITDB	6	N/A	RCNN	98.29	N/A	N/A	99.14
Li et al. [69]	MITDB	-	N/A	CNN	97.96	N/A	N/A	84.94
Chen et al. [70]	MITDB	6	10	CNN + LSTM	99.32	97.75	97.66	N/A
Yıldırım et al. [71]	MITDB	17	10	1D CNN	91.33	83.91	N/A	91.33
He et al. [72]	CPSC	9	30	CNN + LSTM	N/A	N/A	N/A	80.6
Yildirim et al. [73]	MITDB	5	1	DULSTM DBLSTM	99.25 99.39	N/A	N/A	N/A
Yao et al. [74]	CPSC	9	1.5	ResNet + BLSTM-GMP	N/A	80.1	82.6	81.2
Fradi et al. [44]	MITDB, TPB	5	1.496	1D CNN	99.61	N/A	N/A	99
Wang et al. [75]	MITDB	5	N/A	Random forest	92.31	89.98	N/A	N/A
El-Saadawy et al. [76]	MITDB	5	N/A	SVM + PNN	88.7	N/A	N/A	N/A
Sahoo et al. [77]	MITDB	6	N/A	PNN + RBF-NN	99.54 99.89	N/A	N/A	N/A
Khairuddin et al. [78]	MITDB	17	N/A	K-NN	97.30	N/A	N/A	N/A
Proposed	AFDB, MITDB, CUDB, VFDB	3 4 6	2	ResNet-18 ResNet-50 ResNet-18&50	97.21 98.36 94.85	96.49 97.92 94.44	95.54 98.20 93.37	95.96 98.05 94.05

CNN = convolutional neural network, LSTM = long short-term memory, DULSTM = deep unidirectional LSTM, DBLSTM = deep bidirectional LSTM, SVM = support vector machine, BLSTM = bidirectional LSTM, GMP = global maximum pooling, K-NN = k-nearest neighbor, CDF = cumulant derived features, PNN = probabilistic neural network, ANN = artificial neural network, RBF-NN = radial basis function neural network.

**Table 17:** Summary of the AAMI arrhythmia types extracted from the MITDB database.

AAMI Type	No. of Beats
Normal (N)	8980
Ventricular ectopic (V)	7202
Supraventricular ectopic (S)	2758
Fusion (F)	799
Unknown (Q)	8307

**Table 18:** Classification performance acquired with 5-fold cross-validation for Sens, Sp, and F1 score.

Type	CV_1			CV_2			CV_3			CV_4			CV_5		
	Sens	SP	F1	Sens	SP	F1	Sens	SP	F1	Sens	SP	F1	Sens	SP	F1
Mean	96.14%	97.00%	96.56%	96.00%	97.76%	96.84%	95.89%	97.26%	96.55%	97.03%	95.63%	96.31%	96.92%	96.78%	96.84%
STD	4.35%	3.39%	3.79%	4.96%	1.90%	3.38%	4.83%	1.88%	3.33%	2.89%	5.42%	4.15%	2.39%	3.07%	2.64%

**Table 19:** Average classification performances including Acc, Sens, Sp, PPV, F1, and kappa.

CV	Acc	Sens	Sp	PPV	F1	Kappa
Mean ± STD	98.21 ± 0.11%	96.40 ± 0.54%	96.89 ± 0.79%	93.26 ± 2.61%	96.65 ± 0.19%	97.44 ± 0.15%

**Table 20:** Comparison between our approach and existing approaches to AAMI arrhythmia types.

Studies	Classes	Method	Acc (%)	Sens	Sp	PPV	F1
Acharya et al. [15]	5	CNN	94.03	96.71	91.54	N/A	N/A
Oh et al. [79]	5	CNN + LSTM	98.10	97.50	98.70	N/A	N/A
Izci et al. [67]	5	CNN	97.42	N/A	N/A	N/A	N/A
Zhu et al. [80]	5	SVM	97.80	88.83	93.76	N/A	N/A
Aphale et al. [81]	5	CNN	92.73	92.00	91.00	N/A	91.00
Sellami et al. [82]	5	CNN	99.48	96.97	99.87	96.83	N/A
Gan et al. [83]	4	DenseNet-BiLSTM	99.44	95.89	99.32	96.11	95.89
Proposed	5	ResNet-50	98.21	96.40	96.89	93.26	96.65

## Data Availability

This study utilizes the publicly available dataset, from https://physionet.org, accessed on 22 June 2020.
